# Superior Mesenteric Artery Syndrome in Anorexia Nervosa: A Case Report and a Systematic Revision of the Literature

**DOI:** 10.3390/nu16040541

**Published:** 2024-02-16

**Authors:** Elena Bozzola, Mariangela Irrera, Flavia Cirillo, Valeria Zanna, Italo Petrelli, Antonella Diamanti, Ylenia Scire, Jibin Park, Alessandra Marchesi, Maria Rosaria Marchili, Alberto Villani

**Affiliations:** 1 Pediatric Unit, Bambino Gesù Children’s Hospital, IRCCS, 00100 Rome, Italy; mariangela.irrera@opbg.net (M.I.); flavia.cirillo@opbg.net (F.C.); ylenia.scire@opbg.net (Y.S.); alessandra.marchesi@opbg.net (A.M.); mrosaria.marchili@opbg.net (M.R.M.); alberto.villani@opbg.net (A.V.); 2 Child and Adolescent Neuropsychiatry Unit, Bambino Gesù Children’s Hospital, IRCCS, 00100 Rome, Italy; valeria.zanna@opbg.net (V.Z.); italo.petrelli@opbg.net (I.P.); 3 Hepatology Gastroenterology and Nutrition Unit, Bambino Gesù Children Hospital, IRCCS, 00100 Rome, Italy; antonella.diamanti@opbg.net; 4 Medicine and Surgery School, Saint Camillus International University of Health Sciences, 00131 Rome, Italy; u.008375@students.unicamillus.org

**Keywords:** anorexia, children, complication, diagnosis, superior mesenteric artery, superior mesenteric artery syndrome

## Abstract

Background. Superior mesenteric artery syndrome (SMAS) is a rare condition caused by the compression of the duodenum, which may occur in the case of fast weight loss. Currently, the relationship between superior mesenteric artery syndrome and anorexia nervosa is still unclear. The aim of this study is to identify the precocious clinical signs and symptoms of SMAS in patients affected by anorexia nervosa so as not to delay the diagnosis. Methods. We present the clinical case of a young female patient with anorexia nervosa complicated by SMAS. We performed a literature review of SMAS in children affected by anorexia nervosa between 1962 and 2023, according to the PRISMA Extension Guide for Scoping Reviews. Results. Reviewing the literature, 11 clinical cases were described for the pediatric age. The median age at diagnosis was 17 years (ranging from 13 to 18 years). The diagnosis of SMAS may be challenging as symptoms overlap those of anorexia, but it should be kept in mind mostly in cases of post-prandial abdominal pain, anxiety or depression, nausea, vomiting, and weight loss. Conclusion. Even specific clinical symptoms may act as flag tags to drive attention to this rare but potentially fatal condition.

## 1. Introduction

Superior mesenteric artery (SMA) syndrome is a rare condition caused by the compression of the third portion of the duodenum between the aorta posteriorly and the superior mesenteric artery anteriorly. The SMA is surrounded by adipose tissue to create an aortomesenteric angle that varies from 38 to 60°. The obstruction of the duodenum occurs when a loss of mesenteric and retroperitoneal fat decreases the fat plane between the duodenum and the SMA, leading to compression that causes further weight loss and, thus, creates a vicious cycle. The reported symptoms, which include abdominal pain, nausea and vomiting, weight loss, early satiety, and abdominal distension, may overlap those of anorexia nervosa [1,2,3].

This study aims to present the case of a young female patient with anorexia nervosa complicated by superior mesenteric artery syndrome and then explore the relationship between SMA syndrome (SMAS) and anorexia nervosa in pediatric patients through a revision of the literature to highlight precocious clinical signs and symptoms so as to delay the diagnosis.

## 2. Materials and Methods

We report a clinical case of a patient affected by anorexia nervosa and SMAS. We performed a literature review of SMAS in children affected by anorexia nervosa between 1962 and 2023. The clinical manifestations, diagnostic approach, treatment, and outcome were reported and analyzed.

Moreover, we performed a scoping review according to the PRISMA Extension Guide for Scoping Reviews. An electronic search was undertaken on the PubMed database on 12 November 2023. To avoid missing results that may be of note to our revision study, constructing our search in PubMed and Embase, we used all of the important concepts from our basic clinical question, avoiding unnecessary filters. So, the terms “Superior Mesenteric Artery Syndrome” and “Anorexia Nervosa” were used as Mesh Terms, initially together, and then all cases of SMAS in children reported in the literature since 1962 were analyzed in the full text to find cases eventually not included in the previous results.

Studies were marked as eligible for the purpose of this study if they met the following inclusion criteria:

- Full-length articles or reviews of children and adolescents up to 18 years old.
- Full description of the clinical case/cases.

The exclusion criteria were as follows:

- Not pertinent to the field of study.
- Involving adults (>18 years).
- Not presenting a clinical description of the patient.

To prevent bias and errors, two researchers independently read titles and full abstracts to exclude non-pertinent articles. Later, the full text of the screened articles was examined to confirm their inclusion in the study. Disagreements regarding inclusion/exclusion were solved by discussion between researchers. Finally, data were filled in a Microsoft Excel 2023 spreadsheet. Extracted data included participant characteristics, interventions, measures, and results/discussion.

## 3. Results

### 3.1. Case Report

M.B., a Caucasic 13-year-old female affected by anorexia nervosa’s restrictive subtype, presented to the Emergency Unit of Bambino Gesù Children Hospital, IRCCS Rome, Italy, in November 2023 for malnutrition, bradycardia, and hypotension. Her family history was mute for diseases, except for a cousin affected by anorexia nervosa. Her personal medical history was silent as well. She was spontaneously delivered after 40 weeks of physiological pregnancy (at birth, her weight was 3650 g and length 55 cm). Her Apgar score was 9 and 10 at 1 and 5 min after birth. Neonatal screening tests for the identification and early treatment of congenital hypothyroidism, phenylketonuria, and cystic fibrosis were negative. The neonatal period had a normal course. She was breastfed for 9 months and started complementary nutrition and gluten at 6 months of age. M.B. had never been hospitalized before, nor had she undergone surgical procedures. The parents divorced three years previously, after a period of arguments and misunderstandings, sharing custody of the sons. M.B. has a good relationship with the other two twin siblings at 6 years of age. On the contrary, she faced relational difficulties and conflicts with her peers. The family had never been signaled to Social Services for child incur. The eating disorder onset five months before began when she voluntarily restricted her food intake to achieve an intentional weight loss of 20 kg. The patient referred to a story of sexual abuse.

On admission, a physical examination revealed a cachectic state with pale skin, subungual cyanosis, and cold extremities. She had a weight of 27.9 kg, a BMI (body mass index) of 12.2 kg/m^2^, hypotension (blood pressure 89/58 mmHg), and bradycardia (55 beats per minute). Laboratory examinations were unremarkable and compatible with dehydration (azotemia level 23 mg/dL). 

From the first day of hospitalization, the girl presented a deflected mood and an oppositional attitude, refusing food. Psychological interviews highlighted obsessive and polarized ideas on shape and suspiciousness towards others who would like to trick her and make her fat. The presence of either depressive symptomatology of post-traumatic origin (secondary to the referred abuse) or of a dissociative component that proved an obstacle to adherence to medical–nutritional indications required supportive psychiatric drug therapy. The anxiety–depressive dimension was treated with sertraline up to a dosage of 125 mg/day, while psychotic and dissociative dimensions were treated with aripiprazole up to a dosage of 10 mg/day. Six weeks later, antipsychotic therapy was switched from aripiprazole to olanzapine (up to a dosage of 10 mg/day), with a marked improvement in dietary compliance. The patient was also supported through the insertion of en-enteral supplementation via a nasogastric tube and a glucosaline solution via a peripheral vein. 

**Figure 1:**
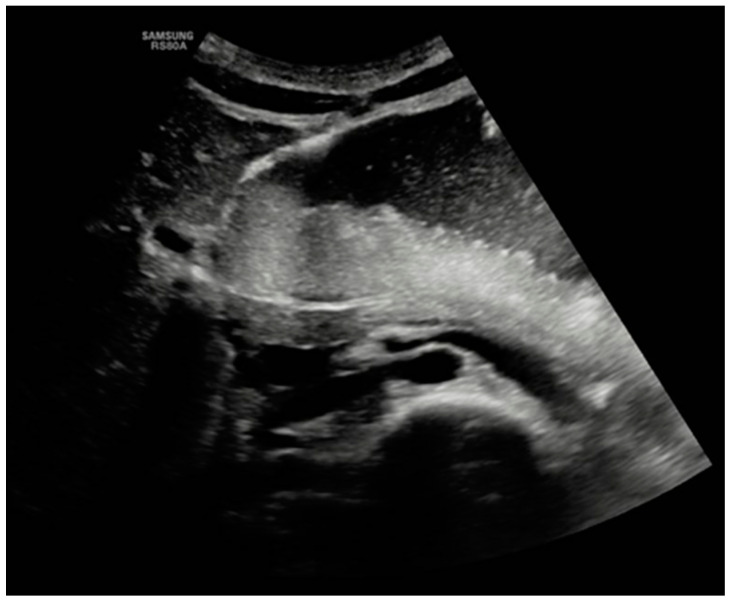
Abdominal ultrasound.

On the third day of hospitalization, the patient began to complain of post-prandial nausea with gastric–biliary vomiting and difficulty urinating. A physical examination revealed a slightly distended abdomen with mild tenderness in the periumbilical area and the left iliac fossa. An urgent abdominal ultrasound evidenced a markedly distended stomach and duodenum and a partially distended bladder (Figure 1).

Enteral nutrition was temporarily suspended, and a bladder catheter was placed, documenting oliguria (output of 150 mL of urine in 24 h). For the persistence of abdominal symptoms, an abdominal X-ray was performed with evidence of gastric air-fluid levels in relation to the over-distension of the stomach up to the left iliac fossa. The patient was evaluated by the surgeon, who hypothesized a picture of upper gastrointestinal obstruction due to SMAS.

A larger nasogastric tube was placed for decompression with a biliary output of 600 mL in one hour; an evacuative enema was performed. Blood exams revealed metabolic alkalosis (pH 7.55, pCO 52, BE 23, HCO_3_ 45) and ipochloremia (87 mEq/L) due to the vomiting. 

A central venous catheter was placed, and parental nutrition was performed, with the resolution of oliguria and normalization of the acid-base balance. 

**Figure 2:**
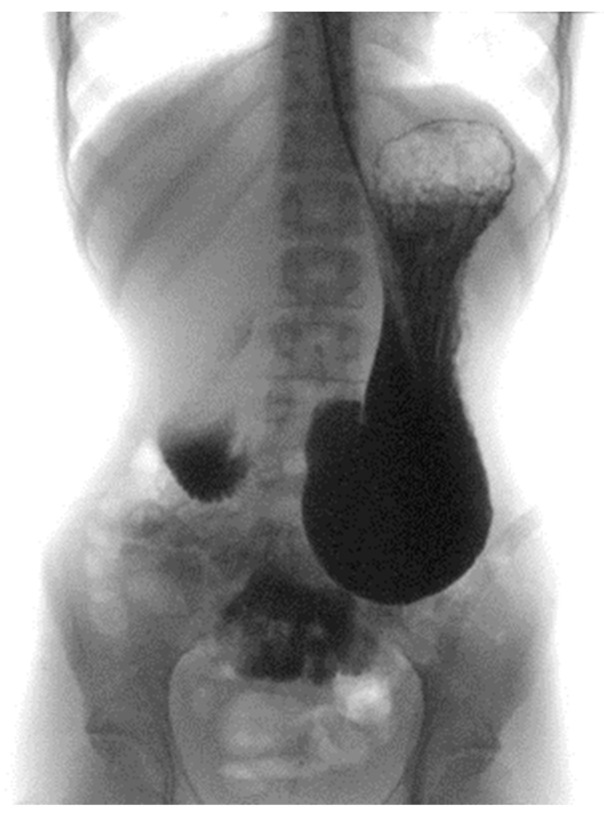
Barium X-ray study.

A barium X-ray study revealed a delayed progression of contrast through the third portion of the duodenum (Figure 2).

**Figure 3:**
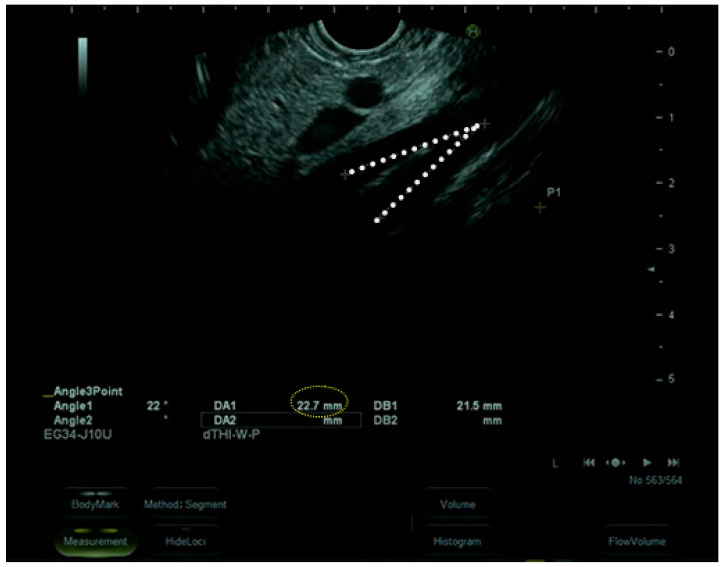
Upper gastrointestinal endoscopy.

The upper gastrointestinal endoscopy excluded the presence of endoluminal masses and revealed that the angle between the superior mesenteric artery and abdominal aorta was 23° with an aorto-mesenteric distance of 3 mm, suggestive of SMAS (Figure 3).

So, to improve nutritional status and to overcome upper gut occlusion, we planned a nutritional strategy that combined and modulated the caloric intake given by parenteral nutrition, enteral nutrition via a naso-jejunal tube, and oral nutrition through small amounts of liquid and semiliquid meals. A multidisciplinary team consisting of pediatrics, psychiatrists, psychologists, and gastroenterologists was involved in the therapeutical approach. The nutritional strategy was defined by the IRCCS Bambino Gesù Children’s Hospital institution protocol for the treatment of Anorexia Nervosa patients, according to NICE guidelines [4]. This strategy is usually based on the combination of natural feeding, oral nutritional supplements, and nasogastric feeding, which is demonstrated as the most effective strategy to achieve weight restoration. We personalized the therapy on the basis of our patient’s needs. On the day of SMAS diagnosis, we planned a combined therapy with tailored parental feeding, enteral feeding, and oral supplementation in order to provide 1800 kcal/die. In detail, 60% of energy intake came from tailored parental feeding with lipid emulsion (Smoflipid^®^, Fresenius Kabi Limited, Cheshire, UK). Precisely, the composition was made of 1000 mL of parental nutrition plus 100 mL (=20 g) of Smoflipid^®^ for a total of 1080 kcal/die. As for the other 40%, energy was supplied by enteral nutrition via a naso-jejunal tube and oral nutrition. In detail, an idrolized hypercaloric formula (Peptamen Junior Advance^®^, 1.5 kcal/mL, Peptamen, Bridgewater, NJ, USA) was administrated at a dose of 180 mL, equal to 270 kcal/die. In addition, oral nutrition was based on small amounts of liquid and semiliquid meals, providing 450 kcal/die. Following the improvement in the patient’s general condition, parental nutrition was progressively reduced to 100 kcal/die, and enteral and oral nutrition were increased accordingly. In one month, parental nutrition was discontinued. Afterward, 600 mL of Peptamen Junior Advance ^®^ was administrated only during the nighttime, providing 900 kcal/die. As for the other 900 kcal, they were supplied by oral nutrition (liquid and semiliquid meals). Day by day, nutritional feeding as well as reduced and oral nutrition increased, so that, in 1 month, the patient was discharged with oral nutrition and supplements (Resource Energy^®^, Columbia, MD, USA). No side effects related to the therapy occurred during the entire period of hospitalization. 

Following the beginning of that combined nutritional treatment, weight progressively improved, solid foods were re-started and well tolerated, and parenteral nutrition was reduced.

Three months later, at discharge, she weighed 39.1 kg and was able to receive full oral nutrition.

The second abdominal X-ray, performed before discharge, showed that gastric air-fluid levels and over-distension had disappeared; therefore, we avoided a further upper gastrointestinal endoscopy.

### 3.2. Literature Review

The search on the selected databases produced n°18 articles and reviews on PubMed and n°3 articles and reviews on Embase. Documents were analyzed to confirm their relevance and eligibility. No duplications were identified by researchers. 

According to PRISMA guidelines, of the identified items, all abstracts were analyzed, 5 records were excluded as irrelevant, and 5 records were excluded as the full text was not available.

**Figure 4:**
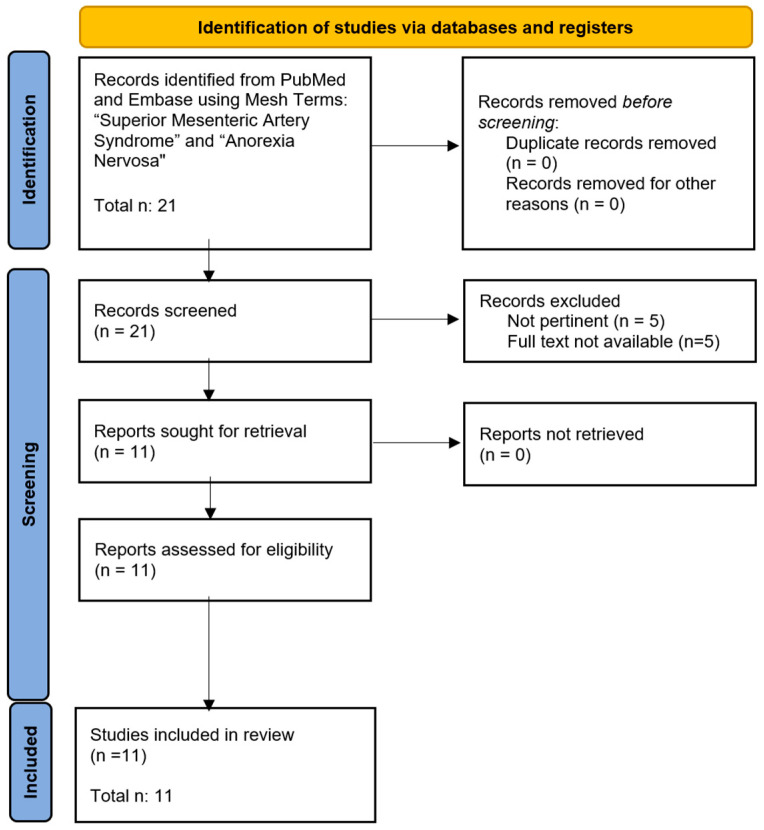
Flow chart of the selected process.

In conclusion, 11 provided records included in the revision [5,6,7,8,9,10,11,12,13,14,15]. Diagram 1 presents the flow chart of the selection process, adapted from PRISMA guidelines (Figure 4).

The included reports were analyzed, and the main highlights are presented in Table 1.

#### 3.2.1. Patient Demography

In total, 12 cases were included in this review as follows: 11 female and 1 male patient (91.7% were female).

The median age at diagnosis was 17 years (ranging from 13 to 18). The weight loss varied from 4 to 35 kg. The median body mass index at diagnosis was 14.1 kg/m^2^, with a range of 8.3–16.4. All of them were affected by anorexia nervosa, and one of them previously experienced sexual abuse.

#### 3.2.2. Complications

The most reported complication (4/12 cases) was acute gastric dilation; one patient reported Nutcracker syndrome and one patient developed pancreatitis [5,6,12,13].

#### 3.2.3. Treatment

All patients received conservative medical treatment (naso-gastric tube, naso-jejunal tube, or parental nutrition), and only in 3 cases out of 12 was surgical treatment necessary.

#### 3.2.4. Outcome

All patients survived after medical and/or surgical management.

## 4. Discussion

According to the literature, SMAS is a rare entity with an estimated prevalence between 0.013% and 0.3%; it is more frequent in young females (91.7%) [16,17,18,19,20,21]. The median age at diagnosis is 17 years (ranging from 13 to 18 years).

Diagnosing SMAS in a restrictive eating disorder is challenging, as symptoms such as vomiting and reduced food intake can be present in anorexia nervosa as well [7]. According to the literature, anorexia nervosa in adolescents presents with a severe restriction of food and energy intake, and the refusal of specific food finalizes in a significant reduction in body weight. Among the characteristics of anorexia nervosa in adolescence, we can include target weights and calorie intake accompanied by a distorted perception of being overweight. Even if they have a normal or low shape, adolescents experience a marked fear of gaining weight or of becoming fat and so behave in certain ways to avoid weight gain. They feel as if they are fat and maintain a distorted self-evaluation of their body and a persistent lack of recognition of the seriousness of their current underweight status. Adolescents frequently follow target levels of excessive exercise or other compensatory behaviors, such as self-induced vomiting or nonprescribed weight-loss products, such as pills, laxatives, and diuretics. Depression, anxiety, and low self-esteem are included among the characteristics of anorexia nervosa [22,23].

There is a two-way relationship between SMAS and anorexia nervosa. At first, weight loss from anorexia nervosa can precipitate SMAS. On the other hand, SMAS can simulate anorexia nervosa because of patients’ food aversion due to obstructive symptoms. So, in several cases of pediatric age, SMAS presents as an anorexia nervosa-like illness, making the differential diagnosis difficult between these two entities [11,24,25,26,27,28,29,30]. In the literature, weight loss ranges from 4 to 35 kg with a median of 16.5 kg. In our case report as well, a young female patient with anorexia nervosa had her hospitalization complicated by superior mesenteric artery syndrome. She was 13 years old and was admitted to the hospital for severe malnutrition (weight loss of approximately 20 kg, BMI 12.2 kg/m^2^).

The anamnesis of being overweight, a young age that does not allow accurate life risk assessment, a restrictive diet and fasting to lose weight, the willingness to control the shape of the body, and the persistent fear of gaining weight make us speculate that anorexia chronologically preceded SMAS. In addition to anorexia-like ideation, M.B.’s history is marked by traumatic events (abuse in the family environment) that would have connoted eating restrictions with particular severity. The girl, who lived in a poorly protective family environment, would, in fact, focus all her attention on an increasingly rapid loss of body weight. The pursuit of hunger and the resulting weight goals had connotations of a self-injuring type: it is possible that the girl sought a way to alienate herself from what was happening in the family through such dysfunctional eating patterns. On the other hand, SMAS worsens eating disorder symptoms, thus interfering with treatment and outcomes.

According to our literature review, the classical presentation of SMAS includes post-prandial abdominal pain (58%), nausea (50%), vomiting (83%), and weight loss (100%). Other symptoms may include abdominal distension (42%) and heartburn (8%). In our case report as well, the patient presented with weight loss and vomiting. These nonspecific symptoms, overlapping with anorexia nervosa, may delay the diagnosis.

Clinically, SMAS may present in an acute or chronic form [6]. The acute form presents with bilious vomiting, abdominal pain, and abdominal distension with the possibility of life-threatening dilatation of the stomach. The chronic form presents post-prandial epigastric pain that is aggravated by oral intake and relieved by emesis, early satiety, anorexia, and weight loss. Epigastric pain is more severe in the supine position and is typically relieved by positioning in the lateral decubitus position (positioning knees to the chest), which relieves tension at the aorto-mesenteric angle. The chronic form leads to inadequate food intake, resulting in severe weight loss and the aggravation of the syndrome. Due to these nonspecific symptoms, SMAS may be misdiagnosed with an eating disorder, leading to a delayed diagnosis [6,19].

According to the literature review, patients with anorexia and SMAS may present with an acute (50%) and chronic form (50%).

It can be noted that patients affected by both anorexia nervosa and SMAS may experience compounding weight loss effects. Diagnosing both diseases is challenging, as presenting symptoms can overlap between the two conditions. Evidence suggests that weight loss due to anorexia nervosa may precipitate SMAS. However, we have to keep in mind that SMAS may develop from other etiologies and can simulate anorexia nervosa as well. Patients may experience vomiting and reduced food intake or food aversion as a palliative measure of obstructive symptoms. In children, SMAS can be the result of a multitude of medical and surgical conditions that lead to weight loss, including tumors, food insecurity, pubertal growth spurts, systemic pathologies, religious purposes, or eating disorders with a voluntary lack of caloric intake [17,31,32,33,34,35,36,37,38]. Psychological comorbidities, such as autism, due to selective feeding can precipitate the syndrome [39,40]. Other underlying aetiologias can include the external compression of anatomic variants, such as a shorter or a higher insertion of the ligament of Treitz or the surgical alteration of the anatomy following spine surgery [41,42].

In effect, superior mesenteric artery syndrome and eating disorders, such as anorexia nervosa, share some common symptoms, making the diagnosis of this serious organic complication more difficult. Patients diagnosed with superior mesenteric artery syndrome report severe abdominal pain, abdominal distension, nausea, and episodes of vomiting following meals [12]. These symptoms, associated with natural weight loss due to difficulty in feeding, are likely to be confused with psychogenic or psychosomatic symptoms, which are typical of anorexia nervosa [10].

Currently, the relationship between superior mesenteric artery syndrome and anorexia nervosa is still unclear. On the one hand, the research points out how superior mesenteric artery syndrome may be the result of a severe lowering in body mass index due both to weight loss and weight maintenance during growth and pubertal development, and on the other hand, other researchers have put forward the hypothesis that anorexia nervosa could instead be a possible consequence of this syndrome. Patients, for example, may develop a psychiatric aversion to food as a defensive formula against the organic difficulties encountered during nutrition and the disabling symptoms experienced after meals [7,14,15].

Furthermore, in some cases present in the literature, it has been highlighted that depressive and anxious symptoms are present during treatments and subsequently during follow-up [5,6,7,15]. These data, in correlation with the risky link between superior mesenteric artery syndrome and anorexia nervosa, suggest the importance of psychological support during and after hospitalization. It is also important to consider the duration of hospitalization, which could have a negative effect on the course of the anorexic disorder [14].

The diagnosis is based on clinical symptoms and supported by the radiological evidence of duodenal obstruction. Criteria for SMAS are formulated on barium X-ray investigations [1,2,43,44]. Recently, alternative imaging modalities have been used to confirm SMAS, such as computed tomography (CT), abdominal ultrasounds (USs), magnetic resonance imaging (MRI), endoscopy, and endoscopic ultrasonography (EUS).

The standard diagnostic modality is a CT scan with intravenous contrast, which allows the measurement of the SMA-Ao angle: the normal mean angle is between 38° and 56°, and the mean aortomesenteric distance is 10–28 mm [42,44].

An aortomesenteric angle less than 25° and aortomesenteric distance less than 8 mm is highly suggestive of SMAS [45,46]. An abdominal US provides a convenient, quick, noninvasive tool to diagnose SMAS [47].

An upper gastrointestinal endoscopy should be prescribed to exclude an intestinal intraluminal obstruction.

In the literature, SMAS in eating disorders has been confirmed using, in most cases, a barium X-ray (36%) or computed tomography (55%) and sometimes by ultrasonography or endoscopy. In our case report, we obtained the final diagnosis as well through endoscopy.

A prompt diagnosis is required to exclude differential diagnoses such as isolated eating disorders, chronic idiopathic intestinal pseudo-obstruction, peptic ulcer disease, functional dyspepsia, cyclic vomiting syndrome, pancreatitis, and gastric outlet obstruction.

In the absence of displacement by an abdominal mass or another pathologic condition that requires immediate surgical exploration, the treatment begins with conservative approaches.

The choice of the type of support varies on the basis of the grade of obstruction and the patient’s tolerance [5]. Nutritional support is generally required in the initial stages of treatment because adequate nutritional support and gastric decompression contribute to the improvement of the symptoms [5,7]. For this purpose, enteral feeding via a nasal tube distal to the obstruction is usually chosen (50%). In the case of gastric and duodenal expansion, gastric decompression may be required. The initial treatment is usually the decompression of the dilated stomach and duodenum via postural change and/or nasal gastric tube suction [5]. Parenteral nutrition is an alternative if the enteral pathway is contraindicated and could even be considered as an adjunct therapy (70%). After decompression therapy, gaining weight to increase the adipose tissue between the SMA and aorta is a resolutive strategy for most cases (75%). If the obstruction is incomplete, first-line treatment is usually nasogastric nutrition or jejunal nutrition; if the obstruction is complete, parenteral nutrition is necessary [48]. The nutrition must be gradual to levitate refeeding syndrome and the electrolyte imbalance. SMAS is typically successfully managed medically. Surgical intervention should be reserved for patients refractory to medical therapy [49,50]. If conservative management fails after 3–4 weeks of therapy, surgical options include open or laparoscopic duodenojejunostomy or duodenal mobilization and the division of the ligament of Treitz [19,43]. In the literature, most patients underwent medical treatment. In detail, 90% required NGT often as a decompressive tool, 50% were treated with nutrition via NJT, and 70% were treated with NP. Just 3 out of 12 cases (25%) required surgical procedures. In our case report as well, the treatment was conservative.

Finally, SMAS in patients affected by eating disorders may be complicated. In the literature, half of the reported cases developed a complication. The most reported complication was acute gastric dilation (33%); one patient reported Nutcracker syndrome and one developed pancreatitis. Three months after the initial diagnosis, our patient did not present with a complication.

## 5. Conclusions

The diagnosis of SMAS should be suspected in young females affected by eating disorders, mostly in the case of a significant and fast loss of weight. Even specific clinical symptoms may act as flag tags to drive attention to this rare but potentially fatal condition. Diagnosis should not be delayed in order to avoid an incomplete therapeutical approach and to prevent complications.

## Figures and Tables

**Table 1:** Cases of SMAS in children affected by eating disorders reported in the literature.

Reference	Sex	Age (Years)	Weight Loss (kg)	BMI (kg/m^2^)	Comorbidities	Complications	Medical Treatment	Surgical Treatment
Case index	F	13	20	12.2	AN Sexual abuse	Acute gastric dilatation	NGT PN NJT	No
1 (Bloomberg L et al.) [7]	F	18	6.8	15.5	AN	No	NGT PN	No
2 (Gwee K et al.) [6]	F	17	10	16.4	AN	Pancreatitis	NGT NJT	No
3 (Osegueda de Rodríguez EJ et al.) [5]	F	17	22.5	8.3	AN	Nutcracker syndrome, Gastro duodenal expansion	NGT PN NJT	Division of the ligament of Treitz and left renal vein. Partial gastrectomy. Duodenal and jejunal anastomosis
4 (Elbadaway MH et al.) [8]	F	18	Unknown	12.7	AN	No	Unknown	Gastrojejunostomy
5 (Sours JA et al.) [9]	F	17	35	Unknown	AN	No	NGT PN	No
6 (Lee Cw et al.) [10]	F	15	4	16.4	AN	No	PN	No
7 (Froese AP et al.) [11]	M	16	Unknown	Unknown	AN	No	Conservative (not specified)	No
8 (Stheneur C et al.) [12]	F	14	17	14.4	AN	Acute gastric dilatation	NGT PN	No
9 (Miyazawa Y et al.) [15]	F	17	16	13.8	AN	No	NGT	Duodenal anterior replacement
10 (Kim T et al.) [13]	F	17	9.2	11.9	AN	Gastric Emphysema	NGT NJT PN	No
11 (Verhoef PA et al.) [14]	F	16	22	15	AN	No	NGT NJT	No
**Reference**	**Acute or Chronic Presentation**		**Symptoms**		**Time from Weight Loss Onset and Hospitalization**		**Radiologic Methods Used in the Diagnosis**	
Case index	Acute		Weight loss, Vomiting		5 months		Upper gastrointestinal endoscopy	
1 (Bloomberg L et al.) [7]	Acute		Weight loss, Abdominal pain, Abdominal distension, Nausea, Vomiting		3 months		Contrast-enhanced abdominal CT	
2 (Gwee K et al.) [6]	Acute		Weight loss, Vomiting, Abdominal distension, Abdominal pain		1 year		Contrast-enhanced abdominal CT	
3 (Osegueda de Rodríguez EJ et al.) [5]	Acute		Weight loss		1 year		Contrast-enhanced abdominal CT	
4 (Elbadaway MH et al.) [8]	Chronic		Weight loss, Heartburn, Abdominal pain, Post-prandial vomiting		4 years		X-ray fluoroscopy	
5 (Sours JA et al.) [9]	Chronic		Weight loss, Nausea, Post-prandial vomiting, Abdominal distension		6 months		X-ray	
6 (Lee Cw et al.) [10]	Chronic		Weight loss, Abdominal pain, Abdominal distension		2 months		Contrast-enhanced abdominal CT	
7 (Froese AP et al.) [11]	Acute		Weight loss, Nausea, Vomiting		13 months		X-ray	
8 (Stheneur C et al.) [12]	Chronic		Weight loss, Nausea, Post-prandial vomiting, Abdominal pain, Abdominal distension		6 months		X-ray	
9 (Miyazawa Y et al.) [15]	Acute		Weight loss, Recurrent vomiting, Abdominal pain		18 months		Ultrasonography Arteriography Endoscopy	
10 (Kim T et al.) [13]	Chronic		Weight loss, Nausea, Post-prandial vomiting		2 years		Contrast-enhanced abdominal CT	
11 (Verhoef PA et al.) [14]	Chronic		Weight loss, Nausea, Abdominal pain, Vomiting		16 months		Contrast-enhanced abdominal CT	

Abbreviations: NGT = naso-gastric tube; NJT = naso-jejunal tube; PN = parental nutrition; BMI = body mass index; AN = anorexia nervosa; CT = cerebral tomography.

## Data Availability

The data presented in this study are available in Bozzola’s study. These data were derived from the following resources available in the public domain: PubMed, internet sources.
